# Inhaled Pharmacotherapy and Stroke Risk in Patients with Chronic Obstructive Pulmonary Disease: A Nationwide Population Based Study Using Two-Stage Approach

**DOI:** 10.1371/journal.pone.0130102

**Published:** 2015-07-09

**Authors:** Hui-Wen Lin, Chi-Li Chung, You Shuei Lin, Chia-Ming Yu, Chun-Nin Lee, Mauo-Ying Bien

**Affiliations:** 1 Department of Mathematics, Soochow University, Taipei, Taiwan; 2 School of Respiratory Therapy, College of Medicine, Taipei Medical University, Taipei, Taiwan; 3 Division of Pulmonary Medicine, Department of Internal Medicine, Taipei Medical University Hospital, Taipei, Taiwan; 4 Department of Physiology, School of Medicine, College of Medicine, Taipei Medical University, Taipei, Taiwan; 5 Department of Neurology, Taipei Medical University Hospital, Taipei, Taiwan; 6 Division of Pulmonary Medicine, Department of Internal Medicine, Wan Fang Hospital, Taipei Medical University, Taipei, Taiwan; 7 Division of Pulmonary Medicine, Department of Internal Medicine, Shuang Ho Hospital, Taipei Medical University, Taipei, Taiwan; Hunter College, UNITED STATES

## Abstract

**Background and Purpose:**

Patients with chronic obstructive pulmonary disease (COPD) are at higher risk of stroke than those without COPD. This study aims to explore the impact of inhaled pharmacotherapy on stroke risk in COPD patients during a three-year follow-up, using a nationwide, population-based study and a matched cohort design.

**Methods:**

The study cohort comprised 10,413 patients who had received COPD treatment between 2004 and 2006; 41,652 randomly selected subjects comprised the comparison cohort. Cox proportional hazard regressions and two-stage propensity score calibration were performed to determine the impact of various inhaled therapies including short-acting muscarinic antagonists, long-acting muscarinic antagonists, short-acting β-agonists (SABAs), long-acting β-agonists (LABAs), and LABA plus inhaled corticosteroid (ICS), on the risk after adjustment for patient demographic characteristics and comorbid disorders.

**Results:**

Of the 52,065 sampled patients, 2,689 (5.2%) developed stroke during follow-up, including 727 (7.0%) from the COPD cohort and 1,962 (4.7%) from the comparison cohort (*p* < 0.001). Treatment with SABA was associated with 1.67-fold (95% CI 1.45–1.91; *p* < 0.001) increased risk of stroke in COPD patients. By contrast, the cumulative incidence of stroke was significantly lower in those treated with LABA plus ICS than those treated without (adjusted hazard ratio 0.75, 95% CI 0.60–0.94, *p* = 0.014).

**Conclusions:**

Among COPD patients, the use of inhaled SABA is associated with an increased risk of stroke, and combination treatment with inhaled LABA and ICS relates to a risk reduction. Further prospective research is needed to verify whether LABA plus ICS confers protection against stroke in patients with COPD.

## Introduction

Chronic obstructive pulmonary disease (COPD), characterized by enhanced airway inflammatory response to noxious gas and persistent airflow limitation [1], is a leading cause of morbidity and mortality globally and leads to a growing and substantial socioeconomic burden [2]. Systemic inflammation is often present in COPD patients and links between COPD and cardiovascular diseases that greatly influence the prognosis, including hypertension, ischemic heart disease and stroke [3, 4]. Moreover, patients diagnosed with COPD are at higher risk of subsequent stroke than those without [5], and the incidence rate of stroke increased significantly following acute exacerbations of COPD [6].

Current COPD guideline advocates that inhaled bronchodilators, including short-acting muscarinic antagonists (SAMAs), short-acting β-agonists (SABAs), long-acting muscarinic antagonists (LAMAs) or long-acting β-agonists (LABAs) are used first for treatment of COPD, and inhaled corticosteroids (ICSs) are added for patients with frequent exacerbations [1]. These agents, either alone or in combination, were reported to have modifying effects on COPD inflammation [7]. Recently, two multi-center, randomized, double-blind, placebo-controlled trials reported that administration of LAMA or LABA plus ICS could provide symptomatic relief, improve lung function and reduce acute exacerbations in patients with COPD [8, 9]. However, to date, the impact of these pharmacological agents on the risk of comorbidities associated with COPD remains unknown.

We thus used a nationwide, population-based dataset to evaluate the effect of various inhaled pharmacotherapy on the risk of stroke among COPD patients during a 3-year follow-up.

## Materials and Methods

### Main Study

A dataset “Longitudinal Health Insurance Database 2005 (LHID2005)” released by the Taiwan National Health Research Institute (NHRI) was used in this study. In order to provide affordable health care for all residents in Taiwan, Taiwan government initiated its National Health Insurance (NHI) program on March 1, 1995. Currently, the program covers over 25 million enrollees, representing around 98% of the population [10]. In cooperation with the Bureau of NHI, NHRI of Taiwan provides all the medical claim data of 1,000,000 beneficiaries, randomly sampled from all 25 million NHI enrollees [10]. As the data set used in this analysis comprised of de-identified secondary data released to the public for investigation purposes, this study was exempt from full audit by the TMU-Joint Institutional Review Board of Taipei Medical University.

### Validation Study

The National Health Interview Survey sampled from the entire Taiwan population was used as an external validation study. The National Health Interview Survey 2005 (NHIS2005) assessed health behaviors and quality of life, and the details of NHIS2005 can be referred to Guo et al [11].

### Study Design and Samples

Study cohort and comparison cohort were both selected from the LHID and subjects aged less than or equal to 50 years were excluded. The study cohort comprised all patients who had visited ambulatory care centers for the treatment of COPD between January 1, 2004 and December 31, 2004. Patients were included in the cohort if they fulfilled all the following criteria: (1) at least 3 consensus COPD diagnoses (ICD-9-CM codes 491, 492, or 496) in the year before treatment initiation (i.e., the date of the first claim for a COPD pharmacotherapy on or after January 1, 2004); (2) no prior diagnosis of stroke in the year before treatment initiation; (3) more than three claims for a COPD pharmacotherapy between January 1, 2004, and December 31, 2004, recorded in the LHID databases; (4) no claim for a COPD pharmacotherapy ≥1 year before treatment initiation to assess the effect of a new use of therapy; (5) no change between COPD pharmacotherapies in the year after treatment initiation. The NIH program covered all the cost of prescribed COPD pharmacotherapy. As a result, the study cohort comprised 10,413 subjects.

The comparison cohort was sampled from the remaining subjects in the LHID2005 with the exclusion of subjects who had been diagnosed as having stroke before 2004 and having COPD diagnosis (ICD-9-CM codes 491, 492, or 496) during 2004 to 2006. There were 41,652 randomly selected subjects (four for each subject in the study cohort) matched with those in the study cohort in terms of gender and age (51–60, 61–70 and > 70 year-old).

### Inhaled Pharmacotherapy

Inhaled COPD medications that had been prescribed to COPD patients were recorded in the LHID databases in five categories: (1) SAMA: Ipratropium bromide metered-dose inhaler (MDI) (Atrovent, Boehringer Ingelheim, Germany); (2) SABA: Fenoterol hydrobromide MDI (Berotec, Boehringer Ingelheim, Germany), Terbutaline sulfate Turbuhaler (Bricanyl, GlaxoSmithKline, UK) or Salbutamol sulfate MDI (Ventolin, GlaxoSmithKline, UK); (3) LAMA: Tiotropium HandiHaler (Spiriva, Boehringer Ingelheim, Germany); (4) LABA: Formoterol Turbuhaler (Oxis, AstraZeneca, Sweden) or Salmeterol MDI (Serevent, GlaxoSmithKline, UK); (5) LABA/ICS: Formoterol/Budesonide Turbuhaler (Symbicort, AstraZeneca, Sweden), or Salmeterol/Fluticasone MDI (Seretide, GlaxoSmithKline, UK). The accumulated prescription for each claim of these inhaled pharmacotherapies must last > 14 days and the use of these inhalers were not mutually exclusive. For patients treated with regular long-acting inhalers (LAMA, LABA or LABA/ICS), a brief use of SABA or SAMA as an on demand rescue for acute exacerbation was allowed. Accordingly, the treatments for COPD patients were categorized as using (1) SAMA, (2) SABA, (3) LAMA, (4) LABA, and (5) LABA/ICS. To assess the precise impact of a particular class of inhaled pharmacotherapy, the switch from the initial inhaler to other category of inhalers during the follow-up was precluded from these categorizations. Moreover, in order to assess the independent contribution of each agent to the stroke risk, patients who used combination inhaler SABA/SAMA: salbutamol/ipratropium bromide MDI (Combivent, Boehringer Ingelheim, Germany) were not included in the analysis.

### Survival Analysis

To avoid the immortal time bias [12], the cohort entry for every COPD patient was taken as the date of the first claim for any of the five inhaled COPD pharmacotherapy, as described in the inclusion criteria. Each subject was then individually followed up to 3 years or until they developed stroke (ICD-9-CM codes 430~432 (hemorrhagic) and 433~435 (ischemic)). The date of final follow-up is December 31, 2006. The median durations of follow-up were 963 days (IQR 709–1077 days) for total subjects, 520 days (IQR 299–725 days) for subjects developing stroke, and 990 days (IQR 713–1077 days) for stroke-free subjects. There are 2,689 first strokes recorded during the follow-up time.

### Statistical Analysis

All statistical analyses were performed using the matched cohort study design and applying the SAS statistical package (SAS System for Windows, Version 9.3) and SPSS. Pearson Chi-square test was used to compare demographic differences between the COPD and comparison cohorts in main and validation studies. Cox model was performed to determine whether subjects with COPD have a higher risk of stroke and whether various inhaled COPD pharmacotherapy affect the risk after adjusting for selected comorbid medical disorders of the sampled patients. The propensity score calibration method was proposed to correct the bias of estimator after adjusted unmeasured confounders. For detail, please refer to next paragraph. The three-year stroke-free survival rate was evaluated by using the Kaplan-Meier method, and the log-rank test was also applied to examine the differences in the risk of stroke between with SABA use and without SABA use, and with LABA plus ICS use and without LABA plus ICS use in COPD cohort. A *p*-value < 0.05 was used to determine the significance of predictors in the models.

### Two-stage Propensity Score Calibration

In order to deal with the unmeasured confounding problem, we applied a two-stage approach. The method of combining samples from primary and validation data for adjusting biases caused by unmeasured confounders has been studied in the literature of two-stage designs [13–15]. We used inclusion criteria consistent to those for the primary study (LHID cohort) to identify 471 patients with COPD and 1896 age- and gender-matched subjects in the comparison cohort between 2004 and 2006 from NHIS2005, including smoking, drinking and BMI variables, which were not observed in our primary study. We then proposed a two-stage method which has been studied by Stürmer et al., to combine with the propensity score to adjust the unmeasured high-dimensional confounders (such as smoking, drinking and BMI) by using the information of NHIS2005 [14].

Let T denote an indicator interesting variable for COPD or medication, take value 1 if patients have COPD, and 0 otherwise. Let C be a vector of observed confounding variables such as patient’s age, sex, diabetes mellitus, hypertension, coronary artery disease, and hyperlipidemia. Let U denote an indicator for unmeasured confounders in our primary study. These include unmeasured confounders for smoking, drinking, and BMI. Let propensity score *PS* = Pr(T = 1|C), we will consider modeling of the estimated PS-stroke association in a Cox proportional hazards model h(t | T, ps) = h_0_ (t) exp {*β*_*T*_T + *β*_*C*_ps} we would define propensity score *P S*_*ep*_ = Pr(*T* = 1|*C*) as the error-prone variable and *PS*_*GS*_ = pr(*T* = 1|*C*, *U*) as the gold-standard in the validation study. The measurement error model then is E(*PS*_*GS*_ | *T*, *PS*_*ep*_) = *γ* + *γ*_*T*_ *T* + *γ*_*C*_ *PS*_*ep*_, then the regression cerebration [16] adjusted C and U confounding factors estimator for the effect of T is $\beta_{T}^{*}\;=\;{\hat{\beta }}_{T}\;-\;\frac{{\hat{\beta }}_{C}}{{\hat{\gamma }}_{C}}\;{\hat{\gamma }}_{T}$.

## Results

### Study Population

Table 1 showed the distributions of socio-demographic characteristics and selected comorbid medical disorders between COPD and comparison cohorts in LHID main Database and in NHIS validation Database. In the main study, 62.4%, 25.8%, 36.9% and 25.6% of the 10,413 COPD patients had comorbidities of hypertension, hyperlipidemia, coronary artery disease and diabetes, respectively. Compared with subjects in the comparison cohort, COPD patients had significantly higher chances to coexist with all these comorbidities. Unmeasured confounders in the main study, including smoking, drinking and body mass index, were provided in the validation database.

**Table 1:** Demographic Characteristics and Comorbid Medical Disorders for Subjects with COPD and in the Comparison Cohort in LHID main Database and NHIS validation Database, 2004–2006.

	Main study				Validation study			
Variable	Subjects with COPD		Comparison Subjects		Subjects with COPD		Comparison Subjects	
	N = 10,413		N = 41,652		N = 471		N = 1896	
	No.	%	No.	%	No.	%	No.	%
Gender								
Male	6848	65.8	27392	65.8	288	61.1	1164	61.4
Female	3565	34.2	14260	34.2	183	38.9	732	38.9
Age (years-old)								
51–60	1868	17.9	7472	17.9	116	24.6	464	24.5
61–70	2853	27.4	11412	27.4	141	29.9	564	29.7
> 70	5692	54.7	22768	54.7	214	45.4	868	45.8
Hypertension								
Yes	6500	62.4	22789	54.7	208	44.2	782	41.2
No	3913	37.6	18863	45.3	263	55.8	1114	58.8
Hyperlipidemia								
Yes	2689	25.8	10121	24.3	89	18.9	301	15.9
No	7724	74.2	31531	75.7	382	81.1	1595	84.1
Coronary heart disease								
Yes	3840	36.9	10374	24.9	99	21.0	251	13.2
No	6573	63.1	31278	75.1	372	79.0	1645	86.8
Diabetes								
Yes	2668	25.6	9990	24.0	83	17.6	298	15.7
No	7745	74.4	31662	76.0	388	82.4	1598	84.3
Smoking								
Yes					163	34.6	584	30.8
No					308	65.4	1312	69.2
Drinking								
Yes					132	28.0	526	27.7
No					339	72.0	1370	72.3
BMI (SD)					23.9 (3.8)		24.1 (3.6)	

*Abbreviation*: COPD = chronic obstructive pulmonary disease

### Survival Analysis

During the three-year follow-up period, there were 2,689 (5.2%) of the 52,065 sampled subjects developed stroke, including 727 (7.0%) of the COPD cohort and 1,962 (4.7%) of the comparison cohort, which were equal to 343, and 192 per 10,000 person-year, respectively (Table 2).

**Table 2:** The Crude and Adjusted Hazard Ratios for Stroke among the Sample Subjects during the Three-year Follow-up Period Starting from the Index Ambulatory Care Visits (N = 52,065).

	Total sample N = 52,065	Comparison subjects N = 41,652	Subjects with COPD N = 10,413
Occurrence of stroke, N (%)	2,689 (5.2%)	1962 (4.7%)	727 (7.0%)
Incidence per 10000 person-year	218	192	343
Crude HR (95% CI)	–	1.00	1.82* (1.67–1.99)
Adjusted HR (95% CI)^a^	–	1.00	1.65* (1.51–1.79)
Propensity score calibration adjusted HR (95% CI) ^b^	–	1.00	1.62* (1.49–1.77)
Adjusted HR (95% CI)^a^ for ischemic stroke		1.00	1.64* (1.49–1.82)
Adjusted HR (95% CI)^a^ for hemorrhagic stroke		1.00	1.18 (0.89–1.57)

*Abbreviation*: COPD = chronic obstructive pulmonary disease; HR = hazard ratio; CI = confidence interval.

^a^Adjustment for patient’s age, gender, hypertension, coronary heart disease, hyperlipidemia and diabetes.

^b^Adjustment for patient’s age, gender, hypertension, coronary heart disease, hyperlipidemia and diabetes and unmeasured confounders including smoking, drinking and body mass index.

*p < 0.001.

Table 2 also showed the crude and adjusted hazard ratios (HR) for stroke between these two cohorts. For COPD subjects, the hazard of stroke was 1.82 times greater than that of the subjects in the comparison cohort (95% CI 1.67–1.99, *p* < 0.001). After adjusting for age, gender, hypertension, hyperlipidemia, coronary artery disease and diabetes, the hazard ratio was 1.65 (95% CI1.51–1.79, *p* < 0.001), comparing to that of subjects in the comparison cohort. In addition, using the NHIS2005 as an external validation study, the two-stage calibration for the association between COPD and stroke demonstrated adjusted HR 1.62 (95% CI 1.49–1.77, *p* < 0.001). Moreover, the adjusted HR of ischemic stroke was 1.64 times (95% CI, 1.49–1.82, *p* < 0.001) greater for COPD patients than for patients in comparison cohort. However, there was no significant difference in the hazards of hemorrhagic stroke between the two groups.

### Inhaled Pharmacotherapy and the Risk of Stroke

**Fig 1:**
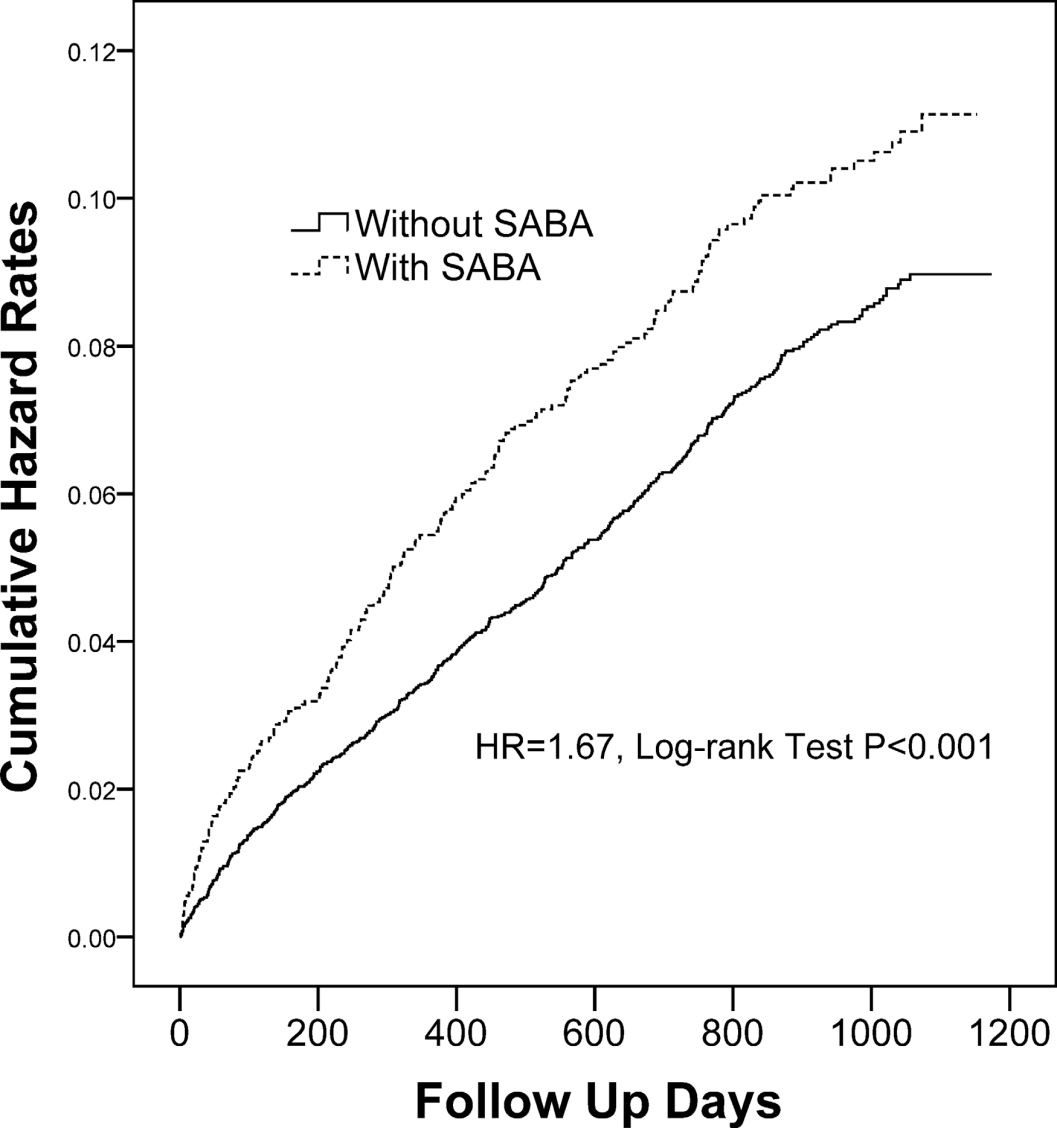
Cumulative incidence of stroke in COPD patients treated with and without inhaled SABA. COPD = chronic obstructive pulmonary disease; SABA = short-acting β-agonist.

**Fig 2:**
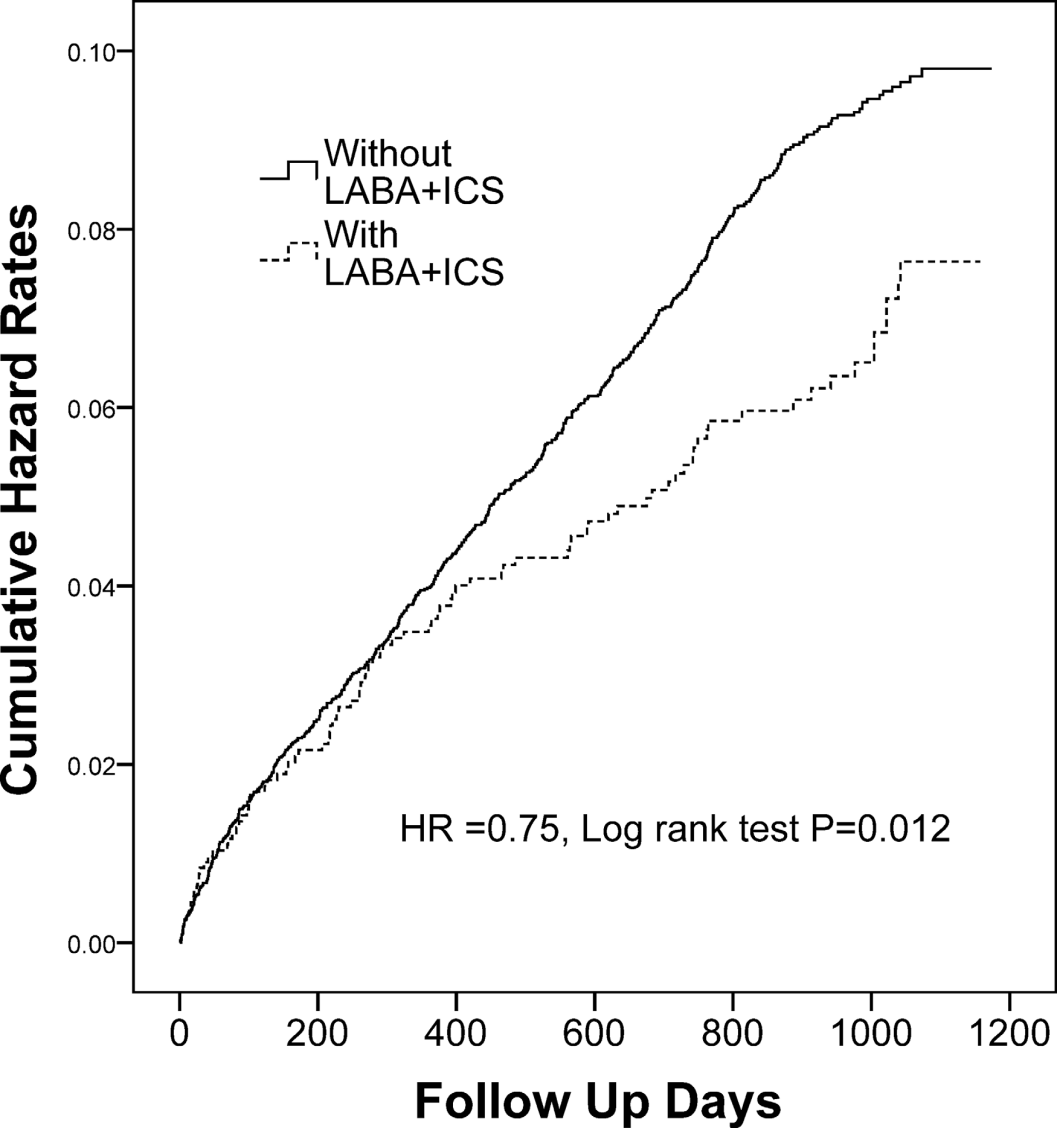
Cumulative incidence of stroke in COPD patients treated with and without inhaled LABA plus ICS. COPD = chronic obstructive pulmonary disease; LABA = long-acting β-agonist; ICS = inhaled corticosteroid.

The adjusted HR for stroke between COPD patients with and without using one of the 5 categories of inhaled pharmacotherapy during three-year follow-up period are shown in Table 3. After adjustment of age, gender, hypertension, hyperlipidemia, coronary artery disease and diabetes, the stroke risk was significantly lower in patients treated with SAMA and higher in patients treated with SABA than those treated without, respectively. However, after two-stage propensity score calibration (Table 4), the impact of SAMA on stroke risk did not reach statistical significance, while the adjusted HR for stroke was increased to 1.67 (95% CI 1.45–1.91, *p* < 0.001) with the use of SABA. Moreover, Table 3 also showed that the stroke risk was significantly decreased in patients treated with LABA plus ICS, as compared to those treated without (adjusted HR 0.77, 95% CI 0.61–0.97; *p* = 0.028), which was further validated by two-stage propensity score calibration (adjusted HR 0.75, 95% CI 0.60–0.94; *p* = 0.014) (Table 4). Consistently, the cumulative incidence of stroke was significantly higher in patients treated with SABA (Fig 1) and less in patients treated with LABA/ICS (Fig 2) than those treated without, respectively. On the other hand, administration with either LAMA or LABA alone has no significant effect on the risk of subsequent stroke (Table 3).

**Table 3:** Adjusted Hazard Ratios for Stroke (Included Ischemic Stroke and Hemorrhagic Stroke) between COPD Patients with or without Using the Five Categories of Inhaled Pharmacotherapy during Three-Year Follow-up (N = 10,413).

Medication	N	Adjusted HR^b^	95% CI	*p*-value
SAMA	2019	0.80	0.64–0.99	0.045
SABA	2345	1.32	1.08–1.62	0.007
LAMA^a^	607	0.92	0.66–1.28	0.640
LABA^a^	118	0.90	0.44–1.82	0.773
LABA plus ICS^a^	1559	0.77	0.61–0.97	0.028

*Abbreviation*: COPD = chronic obstructive pulmonary disease; SAMA = shorting-acting muscarinic antagonist; SABA = short-acting β-agonists; LAMA = long-acting muscarinic antagonist; LABA = long-acting β-agonists; ICS = inhaled corticosteroid; HR = hazard ratio.

^a^A brief use of SABA or SAMA on demand for acute exacerbation is allowed.

^b^Adjustments for patient’s age, gender, hypertension, hyperlipidemia, coronary heart disease and diabetes.

**Table 4:** Adjusted Hazard Ratios for Stroke between COPD Patients with or without Using the Inhaled Pharmacotherapy by Propensity Score Calibration Method.

Medication	N	Adjusted HR^b^	95% CI	*p*-value
SAMA	2019	1.09	0.89–1.33	0.374
SABA	2345	1.67	1.45–1.91	<0.001
LABA plus ICS^a^	1559	0.75	0.60–0.94	0.014

*Abbreviation*: COPD = chronic obstructive pulmonary disease; SAMA = shorting-acting muscarinic antagonist; SABA = short-acting β-agonists; LAMA = long-acting muscarinic antagonist; LABA = long-acting β-agonists; ICS = inhaled corticosteroid; HR = hazard ratio.

^a^A brief use of SABA or SAMA on demand for acute exacerbation is allowed.

^b^Adjustment for patient’s age, gender, hypertension, coronary heart disease, hyperlipidemia and diabetes and unmeasured confounders including smoking, drinking and body mass index.

## Discussion

Our study showed that in age- and gender-matched subjects, the likelihood of any type of stroke and ischemic stroke were 1.62- and 1.64-times, respectively, greater among patients with COPD than those without COPD during the 3-year follow-up after adjustment of other risk factors and two-stage propensity score calibration. Treatment with SABA was associated with 1.67-fold increased risk of stroke, and LABA plus ICS therapy related to reduced stroke hazard by 25%. To the best of our knowledge, this is the first study to investigate the effect of inhaled pharmacotherapy on risk of stroke among COPD patients in the 3 years following a COPD diagnosis, adjusting for demographic features and comorbid medical disorders.

COPD is a persistent airway inflammatory process involving neutrophils, macrophages and elevated levels of interleukin (IL)-1β, IL-6, IL-8 and tumor necrosis factor (TNF)-α [17]. It has been shown that inflammation may spill-over from the lungs to the blood stream, inducing a systemic inflammatory response [17]. Human studies also demonstrated significantly increased serum levels of TNF-α, C-reactive protein (CRP), fibrinogen and alteration of circulating inflammatory cells in COPD patients compared with controls [18]. The spill-over of lung inflammation may cause endothelial dysfunction and generate a procoagulation state, leading to atherothrombosis which could provide a mechanistic link among COPD, atherosclerosis and acute vascular event [17].

Previous reports has demonstrated increased prevalence of stroke among COPD patients, which may be due to shared risk factors such as smoking and low socioeconomic status [3, 4]. In contrast, few studies were identified describing the risk of subsequent stroke in patients with COPD. A recent longitudinal analysis revealed that COPD was associated with 2.8-fold increase in the rate of acute stroke, independent of other risk factors such as sex and smoking status [5]. Another study reported a 1.25-fold increased risk of stroke 1 to 49 days following COPD acute exacerbation [6]. In line with the previous reports [5, 6], our data demonstrated a significant increase in the risk of developing stroke among COPD patients during a 3-year follow-up, after adjusting for age, gender and other comorbidies associated with stroke. All these findings suggest that the COPD-associated systemic inflammation may predispose to atherosclerosis and cerebrovascular events, or that the coexistence of COPD and stroke in individuals may represent different aspects of a single inflammatory syndrome [17]. Nevertheless, the impact of COPD treatment on the risk of acute vascular event has not yet been investigated.

Inhaled β-agonist, either SABA or LABA, relaxes airway smooth muscle by stimulating β_2_-receptors and improve airflow limitation and symptoms in patients with obstructive airway diseases [19]. However, concerns have been raised about the pro-arrhythmic effect of Inhaled β-agonists through adrenergic stimulation [20, 21], which may precipitate cerebrovascular thrombosis and ischemic stroke. Actually, the use of SABA has been reported to increase the risk of cardiovascular event and stroke associated with bronchial asthma [22]. Similar to the previous report [22], our analysis disclosed that SABA did, while LABA did not, increase the risk of subsequent stroke. The differential impact on stroke hazard can be attributed to the fact that LABA imparts greater lung function improvement and less acute exacerbation [8, 9], which may keep COPD patients from stroke-precipitating arrhythmias. However, as patients with severe stages of COPD more frequently develop acute exacerbation that contributes to the risk of stroke [6], whether the increased risk was linked to the use of SABA or to the disease severity remains to be determined. In the present study, two scenarios are possible for patients treated with SABA: (1) SABA alone was the main treatment of a specific group of COPD patients who might have milder severity and less risk of stroke than those treated with other categories (e.g. LAMA, LABA or LABA/ICS), suggesting that SABA directly contributed to the risk of stroke; and (2) SABA was used as a rescue medicine for patients frequently suffering from acute exacerbation, who might have greater disease severity and exacerbation risk, indicating the association between disease severity and the odds of developing stroke. Both conditions might exist in the current study and further studies incorporating the data of disease severity are needed to verify the virtual impact of SABA on stroke risk in COPD patients.

COPD guidelines advocated the use of inhaled long-acting bronchodilators with or without inhaled corticosteroids to treat moderate to severe COPD patients, depending on the combined assessment including airflow limitation, symptoms, and exacerbation history [1]. Two recent large, randomized, double-blind trial demonstrated that inhaled long-acting bronchodilators with or without inhaled corticosteroids decrease exacerbations and enhance health status in COPD patients [8–9], suggesting that inhaled therapies may modulate COPD inflammation. However, the results of previous reports are conflicting regarding whether inhaled pharmacotherapy can diminish systemic inflammation by reducing the spill-over of airway inflammation [19–21]. Pinto-Plata *et al*. found that the circulating CRP level was 20% lower in COPD patients with inhaled corticosteroid treatment than those without [23]. Furthermore, a double-blind placebo-controlled trial demonstrated that withdrawing inhaled steroids increased serum CRP levels and reintroduction of inhaled fluticasone resulted in 50% and 26% decrease in CRP and IL-6 levels, respectively [24]. However, when repeated as a multicenter, randomized trial, no significant changes in CRP or IL-6 were identified in COPD patients treated with inhaled fluticasone or combination of fluticasone/salmeterol [25]. Moreover, the effect of inhaled pharmacotherapy on the risk of inflammation-related vascular events remains unknown. Our study showed by means of Cox regression and considering influential covariates that addition of ICS to LABA correlated with less incidence of stroke in patients with COPD, whereas treatment with SAMA or long-acting bronchodilator (LAMA or LABA) did not affect the occurrence of cerebrovascular events. This data suggests that LABA/ICS combined treatment may modulate systemic inflammation and alter the risk of stroke in patients with COPD. Accordingly, further large-scaled, randomized control trials are mandatory to validate the beneficial effect of inhaled pharmacotherapy on the risk of cerebrovascular comorbidities.

The strength of this study is the setting of population-based data, which facilitates monitoring all cases of COPD and stroke during the follow up. Additionally, the large sample size confers substantial statistical power for recognizing the virtual distinctions between 2 cohorts and the effect of inhaled pharmacotherapy on stroke risk in the 3 years following a COPD diagnosis. Finally, as this study was centered on actual use of drugs, the results are more likely to reflect usual community care. However, this study has a few limitations. First, this study was not a randomized controlled trial, and the levels of COPD severity were not recorded in the data set, which may confound the measured risk of developing stroke between patients using different inhaled therapy. Nevertheless, the important confounding factors including smoking, drinking, and BMI were adjusted by using two-stage calibration approach. Second, the utilization of COPD medication was estimated on prescription claims, which did not entirely reveal how patients virtually use these medications. However, this study cohort included patients who had at least three prescription claims and no switch of inhaled pharmacotherapy within one year, which may imply a substantial adherence with COPD medications in these patients. Finally, in all cases, the diagnoses of COPD were made by physician but not verified by spirometry because of the lack of this element in administrative claims databases.

## Conclusions

In conclusion, this study demonstrated that patients who have a COPD diagnosis were at an increased risk of stroke in the subsequent 3 years. The use of inhaled SABA was associated with an increased risk of stroke, and combination treatment with inhaled LABA and ICS related to a risk reduction. Further prospective research is needed to verify whether LABA plus ICS treatment confers protection against stroke in patients with COPD.
